# Intracellular ROS mediates gas plasma-facilitated cellular transfection in 2D and 3D cultures

**DOI:** 10.1038/srep27872

**Published:** 2016-06-14

**Authors:** Dehui Xu, Biqing Wang, Yujing Xu, Zeyu Chen, Qinjie Cui, Yanjie Yang, Hailan Chen, Michael G. Kong

**Affiliations:** 1 Centre for Plasma Biomedicine, Xi’an Jiaotong University, Xi’an, Shaanxi 710049, P.R. China; 2 State Key Laboratory of Electrical Insulation and Power Equipment, Xi’an Jiaotong University, Xi’an, Shaanxi 710049, P.R. China; 3 Department of Cardiovascular Medicine, First Affiliated Hospital of the Medical School, Xi’an Jiaotong University, Xi’an, Shaanxi 710049, P.R. China; 4 Frank Reidy Center for Bioelectrics, Old Dominion University, Norfolk, VA, 23508, USA; 5 Department of Electrical and Computer Engineering, Old Dominion University, Norfolk, VA, 23529, USA

## Abstract

This study reports the potential of cold atmospheric plasma (CAP) as a versatile tool for delivering oligonucleotides into mammalian cells. Compared to lipofection and electroporation methods, plasma transfection showed a better uptake efficiency and less cell death in the transfection of oligonucleotides. We demonstrated that the level of extracellular aqueous reactive oxygen species (ROS) produced by gas plasma is correlated with the uptake efficiency and that this is achieved through an increase of intracellular ROS levels and the resulting increase in cell membrane permeability. This finding was supported by the use of ROS scavengers, which reduced CAP-based uptake efficiency. In addition, we found that cold atmospheric plasma could transfer oligonucleotides such as siRNA and miRNA into cells even in 3D cultures, thus suggesting the potential for unique applications of CAP beyond those provided by standard transfection techniques. Together, our results suggest that cold plasma might provide an efficient technique for the delivery of siRNA and miRNA in 2D and 3D culture models.

Cell transfection of genes and exogenous molecules such as therapeutic agents into individual mammalian cells is among the most important technologies utilized in modern molecular cell biology research. Gene delivery methods are usually divided into two major groups: viral and nonviral. Viral vectors, including retrovirus, adenovirus, adeno-associated virus (AAV) and others, generally have a higher transfection efficiency. However, virus transfection poses problems of immunogenicity with virus-mediated host immune reactions and safety risks of integration of viral genes into the host cell genome^1^,^2^,^3^. Among nonviral-based approaches, cationic lipid-mediated gene transfer or lipofection was widely used in biological research, yet these methods are known to be relatively cytotoxic or inefficient at delivering exogenous molecules into cells, especially in primary and stem cells^4^,^5^,^6^,^7^. New technologies, such as bioreducible nanocarriers, have shown promise for improved efficiency and reduced cytotoxicity^8^,^9^,^10^. Electroporation is an efficient and convenient approach for cell transfection that uses the application of electric pulses^11^; however, this method requires special equipment and the cell viability after electroporation is relatively low due to the administered electric shock^12^,^13^,^14^. Boukany *et al*. developed a nano-channel electroporation (NEP) and a novel 3D NEP system device that could deliver transfection agents into individual living cells without affecting cell viability^15^,^16^. Zu *et al*. reported optimization of the electroporation performance with better DNA delivery efficiency and higher cell viability by adding highly conductive gold nanoparticles (AuNPs) to the electroporation solution^17^. In addition to the above major transfection methods, new technologies have been applied to cell transfection. Arita *et al*. reported a laser-simulated gold nanoparticle for single cell transfection by laser-induced breakdown (LIB)^18^, and Ren *et al*. utilized ultrasound energy to study the synergistic effects of ultrasound-targeted microbubble destruction (UTMD) and TAT peptides on gene transfection^19^. On the other hand, cold atmospheric plasma (CAP),a novel technology developed in recent years^20^, has been used in numerous biological applications for the production and delivery of various reactive oxygen species (ROS) and reactive nitrogen species (RNS)^21^,^22^. Examples of its translation into major healthcare innovations include tissue ablation and coagulation, disinfection of food and living tissues, wound healing, and even cancer treatment^23^,^24^,^25^,^26^. Several groups have reported that plasma jet with pulsed high frequency could increase the cell permeability and delivery of GFP vectors in mammalian cells such as HeLa cells, CHL cells, Jurkat cells, Hacat cells and MCF-7 cells^27^,^28^,^29^. Leduc *et al*. reported that plasma jet with pulsed radiofrequency (13.56 MHz) power could induce temporary cell permeabilization and allow macromolecules with a maximum radius below 6.5 nm to enter into HeLa cells^30^. They showed that no degradation of DNA occurred when plasmid DNA suspended in the culture media was treated with the plasma under the same conditions. However, Sakai reported that the plasma could induce DNA strand breaks in a PBS solution^27^. A recent study showed that by using the cell-solution electrode, plasma jet could improve cell permeability compared with the conventional diffusion type plasma^31^. Some publications have discussed transdermal drug delivery by cold atmosphere plasma^32^,^33^,^34^. For cellular transfection enabled by CAP, little has been reported in the current literature about the potential mechanisms and the possibility of successful transfection of cells in 3D cultures. 3D culture models are artificially created environments wherein cells can grow or interact with their surroundings in all three dimensions, which might accurately mimic the status of cells *in vivo*^35^,^36^,^37^,^38^. However, one limitation of the 3D model is that cells are growing in the Matrigel matrix medium, which makes transfection by traditional lipofection techniques or electroporation difficult. A common strategy is to transfect 2D cells in advance and transfer them for further 3D culture^39^, which limits the time points for transfection in the 3D model. Therefore, in this study, we first compared the uptake efficiencies and cell viabilities of the two most commonly used methods, namely liposome-mediated transfection and electroporation to plasma-transfection. Second, we analyzed the relationship of intracellular ROS accumulation and Ca^2+^ influx by plasma as a possible mechanism of cell transfection. Finally, we investigated, for the first time, the possibility of plasma transfection for delivering siRNA and miRNA in a 3D cell culture, which presents a potential application of the plasma in 3D cell transfection.

## Results

**Figure 1:**
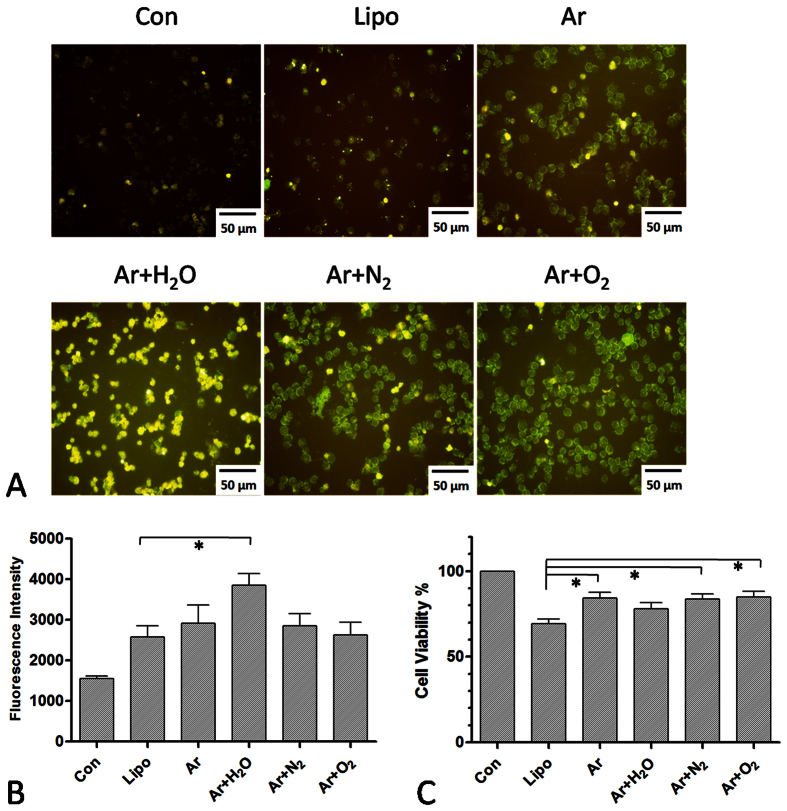
Plasma transfection of DNA-FITC into LP-1 cells. (**A**) Fluorescence image of LP-1 cells 2 h after transfection using various plasmas for 20 s. (**B**) Fluorescence intensity of plasma transfection compared to lipofection (Lipo) (**C**) Corresponding cell viability measured using Cell-Titer-Glo after plasma transfection and lipofection. n = 3, *indicates P < 0.05.

In this study, we analyzed cold atmospheric plasma to determine its efficacy as a transfection vehicle for mammalian cells. Various plasmas were produced at 10 kHz/10 kV. A gas flow of 2 SLM Ar was used as the working gas, coupled with small amounts of functional gases, such as O_2_, N_2_ and H_2_O. The distance between the plasma jet and the medium was fixed at 1.5 cm. Changes in the chemical composition of the gas may increase ROS production and decrease plasma intensity or even stop plasma generation. We tested several gas mixing ratios. We tested an O_2_ admixture at 0%, 0.2%, 0.5% and 1.0%; an N_2_ admixture at 0%, 0.5%, 1.0% and 2.0%; and an H_2_O admixture at 0%, 0.5%, 1.0% and 2.0%. Based on the plasma intensity and stability (monitored using electric current), we established that the optimal ratio for the generation of plasma was Ar + O_2_ (0.5%), +N_2_ (0.5%) and +H_2_O (1%). LP-1 cells were used for plasma treatment and DNA-FITC, exogenous small DNA molecules, were added as a fluorescent marker. After treatment with the various plasmas for 20 s, fluorescence could be detected after 24 h by a fluorescent microscope (Fig. 1A). Compared to lipofection (Lipo), Ar + H_2_O plasma showed a better effect on cellular uptake (Fig. 1A), whereas there was no improvement in cell viability (Fig. 1B,C). Although Ar, Ar + N_2_ and Ar + O_2_ plasma resulted in a similar fluorescence intensity to that of lipofection (Fig. 1B), the level of cell death was lower than that caused by lipofection (Fig. 1C).

**Figure 2:**
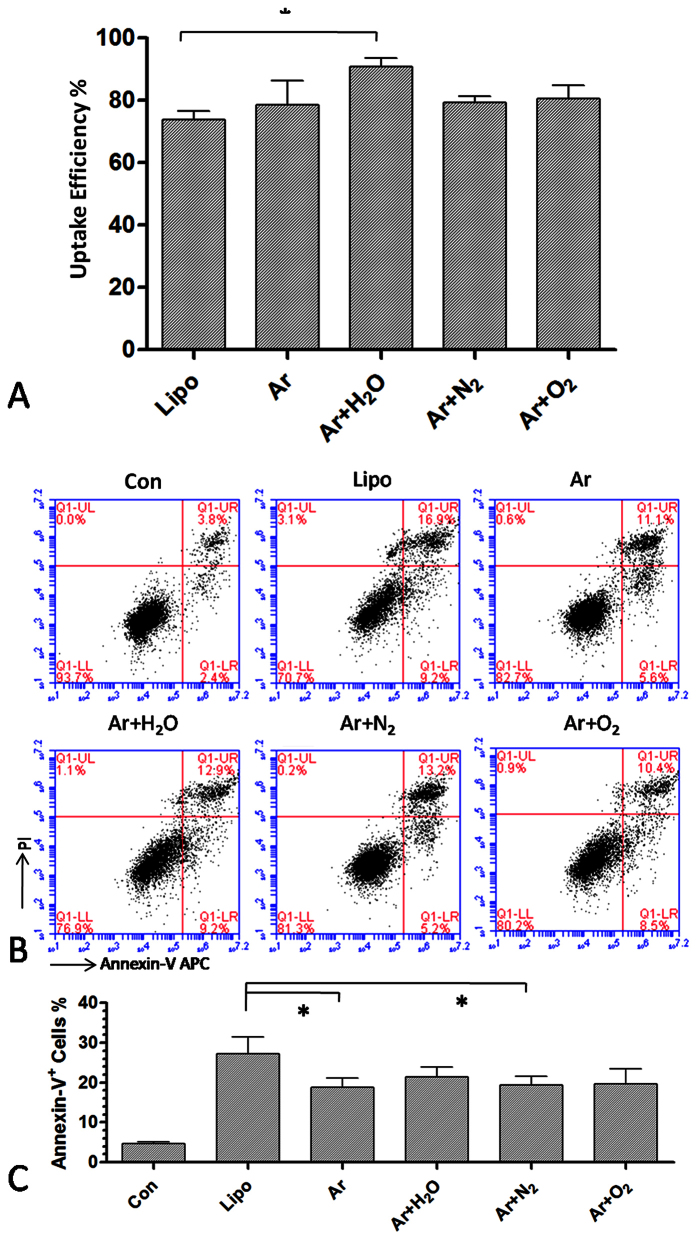
Uptake efficiency and cell apoptosis after plasma and lipofection transfection. (**A**) Uptake efficiency measured by flow cytometry 24 h after plasma transfection for 20 s and lipofection. (**B**,**C**) Cell apoptosis measured by Annexin-V/PI staining 24 h after plasma transfection for 20 s and lipofection. (**C**) shows the percentage of Annexin-V+ cells in each group. n = 3, *indicates P < 0.05.

**Figure 3:**
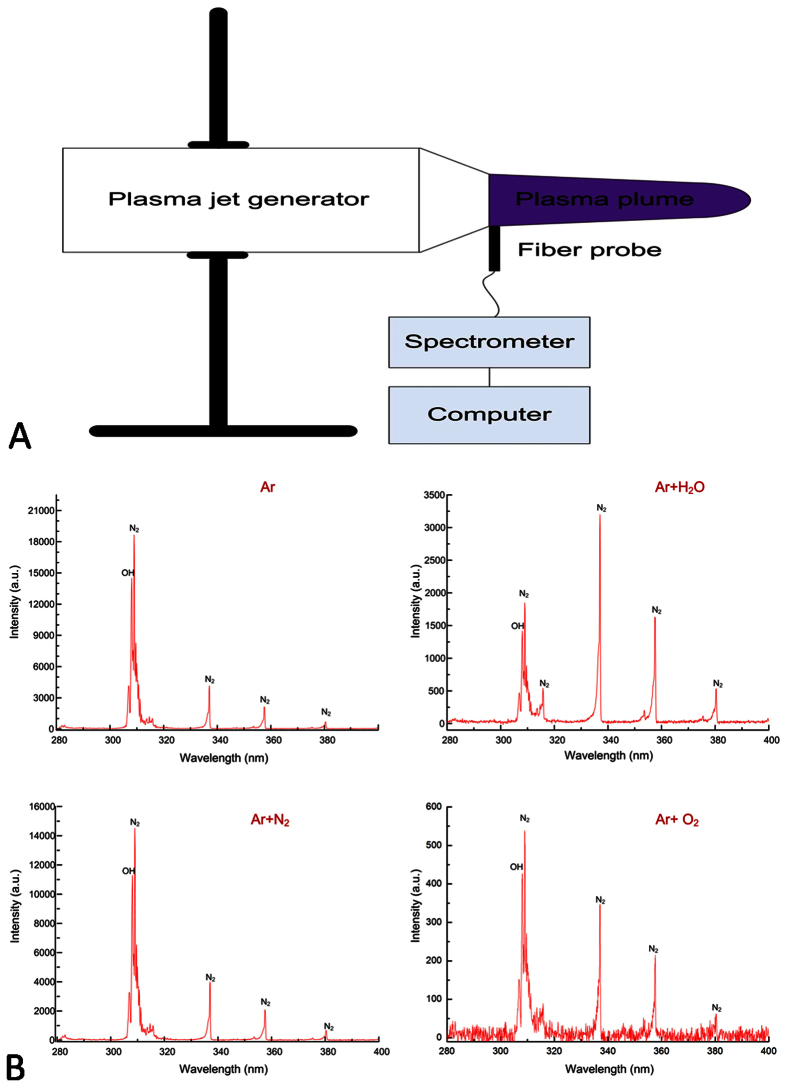
Emission spectra of different plasmas as detected by spectroscopy. (**A**) Schematic of the spectroscopy experimental setup. (**B**) The emission spectra of Ar, Ar + H_2_O, Ar + N_2_ and Ar + O_2_ plasmas are shown; certain unique spectral lines (OH, N_2_) are marked.

**Figure 4:**
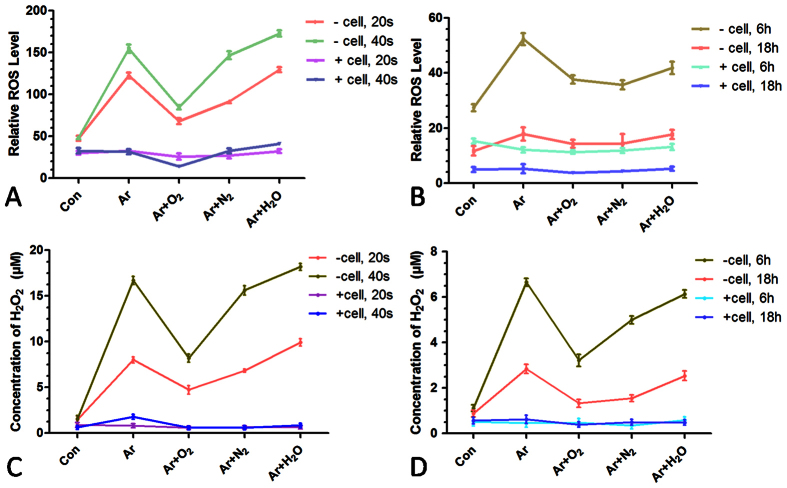
General ROS level and H_2_O_2_ concentration in the liquid after various plasma treatments. (**A**) Detection of general ROS generation in the medium with or without cells immediately after plasma treatments for 20 s and 40 s. (**B**) Detection of ROS generation in the medium 6 h and 18 h after plasma treatments for 20 s. (**C**) Concentration of H_2_O_2_ in the medium with or without cells immediately after several plasma treatments for 20 s and 40 s. (**D**) Concentration of H_2_O_2_ in the medium 6 h and 18 h after plasma treatments for 20 s. n = 3.

We further confirmed cellular uptake efficiency and cell apoptosis after lipofection and plasma transfection by flow cytometry. This experiment demonstrated that Ar + H_2_O plasma transfection had the highest uptake efficiency, up to 90%. Transfection using other plasmas (Ar, Ar + N_2_ and Ar + O_2_) showed similar uptake efficiencies (75–80%) to that of lipofection (Fig. 2A). Annexin-V and PI staining showed that Ar and Ar + N_2_ plasma transfection resulted in fewer apoptotic cells than lipofection (Fig. 2B,C). To understand the effects of different plasmas on cellular uptake in more detail, we used spectroscopy to detect the compounds in the plasma in the vertical direction of the plasma plume (Fig. 3A). Ar, Ar + N_2_ and Ar + O_2_ showed similar spectral line patterns (Fig. 3B). Moreover, the addition of H_2_O, N_2_ or O_2_ reduced the optical emission, suggesting weakening of plasma compared to Ar plasma alone, as we used the same voltage setting (Fig. 3B). Therefore, we further investigated aqueous ROS generation after different plasma treatments. General ROS generation was measured by CM-H2DCFDA following the protocol described in the literature^40^,^41^. The temperature of the medium after different plasma treatments was 25–30 °C. The ROS level in the medium without cells was elevated immediately after the treatment with different plasmas (Fig. 4A) and gradually decreased after 6 h and 18 h (Fig. 4B). In the medium with cells, the general ROS level remained stable despite the plasma treatment (Fig. 4A,B). Similar results were obtained for the long-lived H_2_O_2_ concentration produced by plasma treatment (Fig. 4C,D), suggesting that cells may interact with aqueous ROS and consume the extracellular ROS in the medium. Considering both uptake efficiency and cell viability, we chose to use Ar for plasma transfection in the subsequent studies.

**Figure 5:**
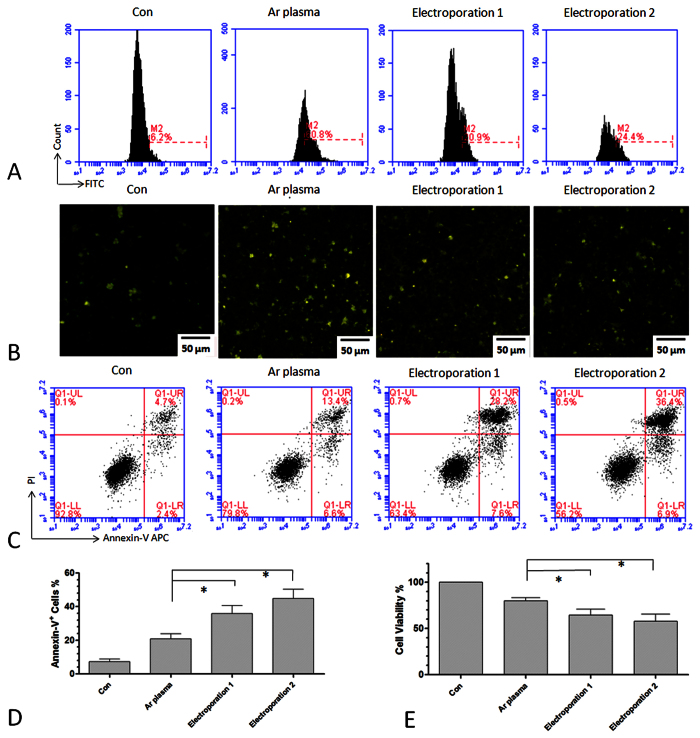
Uptake efficiency and cell apoptosis after plasma transfection and electroporation. (**A**) Uptake efficiency measured by flow cytometry 24 h after Ar plasma transfection for 20 s and two conditions of electroporation. M2 labeled the fluorescence positive cells according to the control group. (**B**) Fluorescence images of LP-1 cells 24 h after Ar plasma transfection for 20 s and electroporations. (**C**,**D**) Cell apoptosis measured by Annexin-V/PI staining 24 h after Ar plasma and electroporation. (**E**) Cell viability measured by Cell-Titer-Glo 24 h after Ar plasma and electroporation. n = 3, *indicates P < 0.05.

Next, we compared plasma transfection with electroporation by analyzing the respective uptake efficiency, cell viability, and apoptosis. Here, we used two different conditions for electroporation: 1) electroporation 150 V, 10.0 ms pulse length, 1 pulse number, and 2 mm cuvette; and 2) electroporation 160 V, 500 μF capacitance, ∞ resistance, and 2 mm cuvette. As shown in Fig. 5, electroporation of DNA-FITC had a lower uptake efficiency than plasma transfection, as determined by flow cytometry (Fig. 5A) and fluorescence microscopy (Fig. 5B). Both conditions for electroporation resulted in a higher degree of cell apoptosis than that of plasma transfection, as measured by Annexin-V and PI staining (Fig. 5C,D). Cell viability after electroporation was also lower than that following plasma transfection (Fig. 5E).

**Figure 6:**
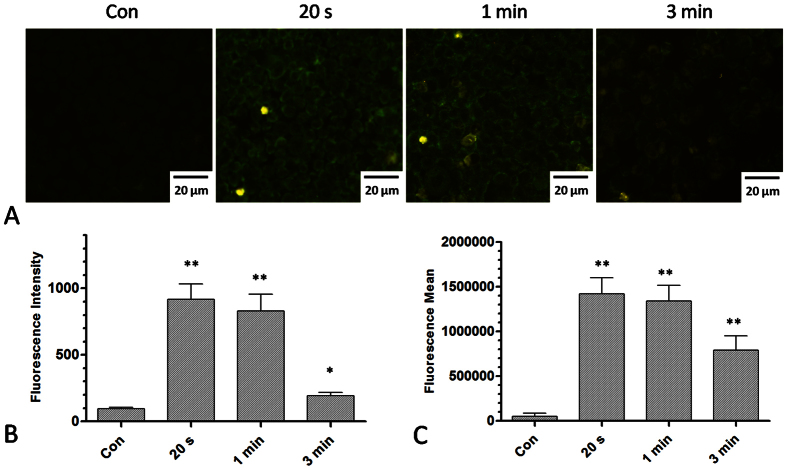
Detection of intracellular ROS levels after plasma treatment for different durations. (**A**) Fluorescence images of DCFH-DA staining after plasma treatment for 20 s, 1 min, and 3 min. (**B**) Fluorescence intensity after plasma treatment measured by fluorescence microscope. (**C**) Fluorescence intensity after plasma treatment measured by flow cytometry. n = 3, *indicates P < 0.05. **indicates P < 0.01.

Because plasma could produce various ROS, which might increase membrane permeability, we first analyzed whether the level of ROS in cells was increased by plasma treatment. Using the fluorescent probe DCFH-DA, we detected the intracellular ROS level after different durations of plasma treatment. Fig. 6A shows the fluorescence images of cells after plasma treatment for 20 s, 1 min and 3 min. These data demonstrate that the intracellular ROS level was increased by plasma treatment, as measured both by fluorescence microscopy (Fig. 6B) and by flow cytometry (Fig. 6C).

**Figure 7:**
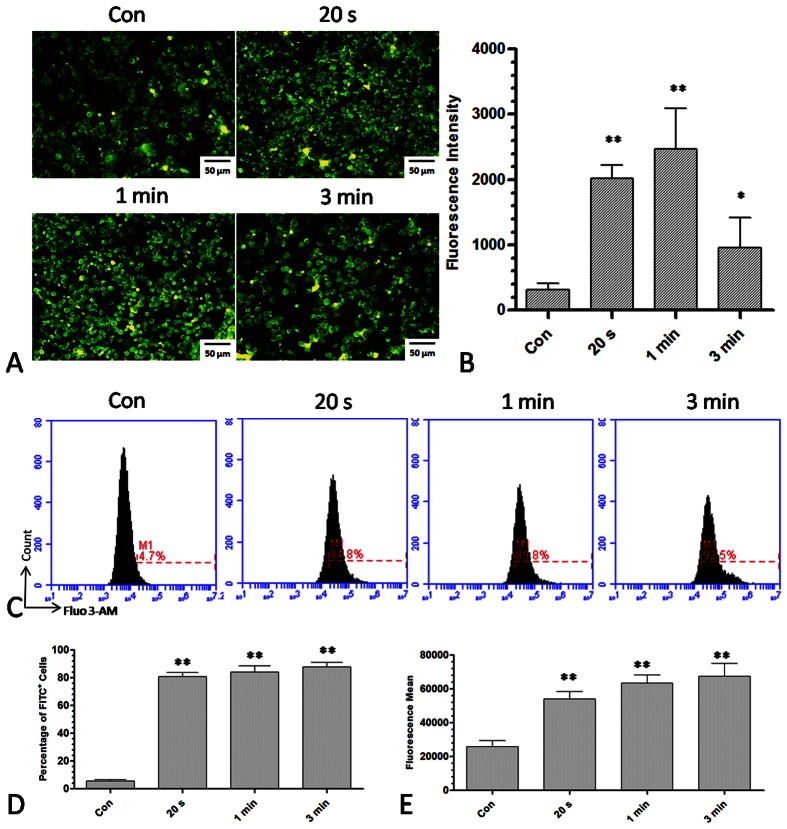
Detection of intracellular Ca^2+^ levels after plasma treatment for different durations. (**A**,**B**) Fluorescence image and intensity of intracellular Ca^2+^ levels stained with Fluo 3-AM after plasma treatment for 20 s, 1 min and 3 min. (**C**) Measurement of the intracellular Ca^2+^ level by flow cytometry after plasma treatment for 20 s, 1 min and 3 min. M1 labeled the fluorescence positive cells with respect to the control group. (**D**,**E**) Percentage of fluorescence positive cells and fluorescence intensity after plasma treatment for 20 s, 1 min, and 3 min. n = 3, *indicates P < 0.05. **indicates P < 0.01.

Next, we wanted to determine whether plasma-induced intracellular ROS accumulation could increase cell membrane permeability. Intracellular Ca^2+^ levels were measured to determine cell membrane permeability. Under normal conditions, the extracellular Ca^2+^ levels are much higher than the intracellular Ca^2+^ levels because Ca^2+^ ion channels are able to pump Ca^2+^ out of the cell through the cell membrane. Under conditions that increase membrane permeability, Ca^2+^ will flux into the cells through the membrane. Fluorescence microscopy showed that the level of intracellular Ca^2+^ was increased after plasma treatment for 20 s and 1 min and that it was decreased at 3 min. (Fig. 7A,B). Flow cytometry data (Fig. 7C) indicate that different durations of plasma treatment could increase the intracellular Ca^2+^ level, as measured by the percentage of the fluorescence positive cells (Fig. 7D) or by the average mean of the fluorescence (Fig. 7E).

**Figure 8:**
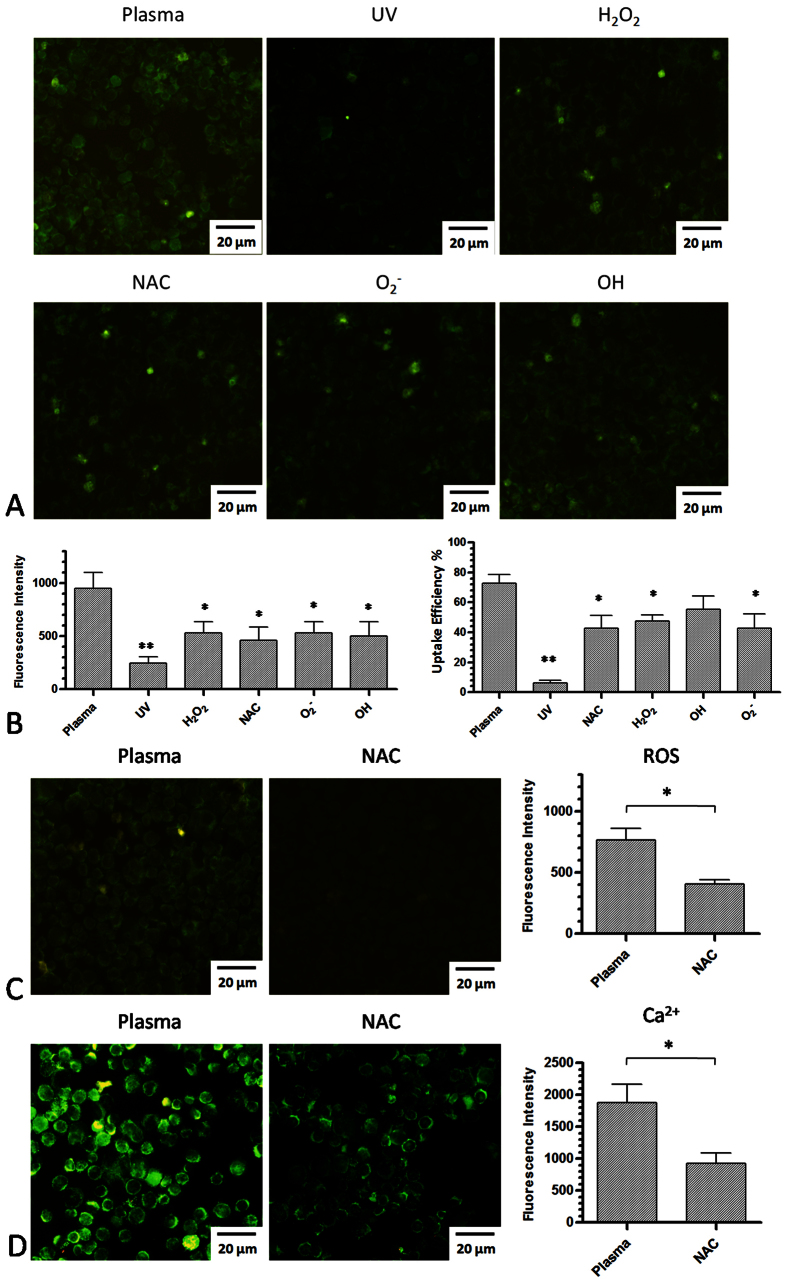
The effect of different ROS scavengers on plasma-transfection, ROS generation, and Ca^2+^ concentration. (**A**) The effect of different ROS scavengers on plasma-transfection. UV indicates that only UV is available to affect cells as all other reactive species were removed by a quartz glass plate. (**B**) Measurement of fluorescence intensity and uptake efficiency by flow cytometry after adding different ROS scavengers. (**C**) The effect of ROS scavenger NAC on intracellular ROS generation. (**D**) The effect of ROS scavenger NAC on Ca^2+^ influx induced by plasma-transfection. n = 3, *indicates P < 0.05. **indicates P < 0.01.

Furthermore, to determine which compound in the plasma might be the major factor that increases cell membrane permeability, we used several different ROS scavengers mentioned in the method section. No reduction of cell viability was found 24 h after incubation with individual scavenger at the working concentration (data not shown). This analysis showed that UV alone yielded few fluorescent cells (Fig. 8A) with a very low uptake efficiency, whereas depletion of other ROS partially reduced fluorescence intensity and the plasma uptake efficiency (Fig. 8B). However, no significant difference was observed between these unique scavengers and the general ROS scavenger NAC. To further confirm that ROS production by the plasma is one of the factors that increase cell membrane permeability to benefit plasma-transfection, we determined the intracellular ROS levels and the intracellular Ca^2+^ levels while adding NAC during treatment of cells with plasma for 20 s. The intracellular ROS level decreased when NAC was added to the medium (Fig. 8C). In addition, the intracellular Ca^2+^ level was also decreased by NAC (Fig. 8D), indicating that NAC could prevent the generation of intracellular ROS and protect the cell membrane.

**Figure 9:**
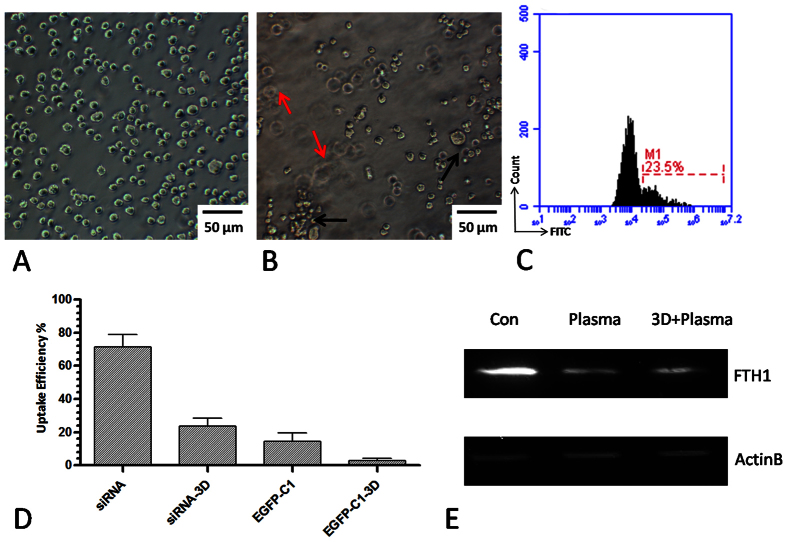
Transfection of siRNA-FITC, miRNA and EGFP plasmid by plasma-transfection in 2D and 3D cultured cells. (**A**) LP-1 cells cultured in the normal 2D condition. (**B**) LP-1 cells cultured in the 3D condition. Black and red arrows indicate different levels of cells in the 3D condition. (**C**) Uptake efficiency of DNA-FITC by plasma-transfection in the 3D condition by flow cytometry. (**D**) Uptake efficiency of siRNA-FITC and pEGFP-C1 in 2D and 3D culture conditions by flow cytometry. n = 3. (**E**) Western blot analysis of FTH1 expression 48 h after transfection of miRNA in 2D and 3D conditions by plasma. n = 3.

To explore the applications of plasma transfection, we also attempted to transfect other oligonucleotides such as siRNA, miRNA, and a large EGFP-C1 plasmid (4.7 kb) in the 2D and 3D culture systems using plasma-transfection. Fig. 9A shows LP-1 cells cultured in normal 2D conditions, whereas Fig. 9B illustrates cells cultured in 3D conditions. In this 3D culture model, the matrix is approximately 2 mm thick and at least 3–4 levels of cells could be observed with the microscope. Meanwhile, the extracellular matrix was also supported for essential signaling activation. Here, we showed that plasma exhibits a good potential for transfecting oligonucleotides directly in 3D cultured cells. As shown in Fig. 9C, DNA-FITC could be transfected into cells even when they were cultured in the 3D condition, although the uptake efficiency was lower than that obtained in the normal conditions, as determined by flow cytometry (Fig. 9C). Small RNA molecules such as siRNA-FITC could also be transfected into cells in 2D or even in 3D conditions. However, large DNA molecules such as pEGFP-C1, showed a relatively poor transfection efficiency in 2D and 3D conditions (Fig. 9D), suggesting that molecule size might be one of the elements that affects the plasma transfection outcomes. We further transfected an miRNA targeting the *FTH1* gene by plasma in 2D and 3D culture conditions and detected the protein expression by western blot after 48 h (Fig. 9E). We confirmed that plasma transfection could be used in biological experiments for transfection of oligonucleotides such as siRNA and miRNA in 2D or 3D culture conditions.

## Discussion

In this study, we demonstrated an effective method for cell transfection using cold atmosphere plasma, which is a mixture of discharged gases. We found that plasma could efficiently deliver oligonucleotides into cells by increasing cell membrane permeability. Chouinard-Pelletier *et al*. reported that using a high pressure inert gas could transiently transfer plasmid DNA and molecules up to 45 kD into HeLa cells^42^ and that this process was mainly mediated by the mechanical force to the cells. In our studies, we observed little effect on the uptake and viability when using only gas flow as a control. This discrepancy in the findings may be due to the much lower flow rate and the dynamic pressure than those in the previous study. In our study, we observed a high uptake efficiency (70–90%) of DNA-FITC, whereas the efficiency of GFP vector in LP-1 cells was less than 13%. Sakai etal. reported a transfection efficiency of approximately 20% for GFP^27^. The small amount of liquid for plasma treatment (200 μL in 60 mm dishes) used in their study may account for this difference in the results. Cells were directly exposed to plasma flow and charged particles, which may contribute to the transfection but result in cell damage as even a few seconds of plasma treatment will induce cell death. In addition, the GFP fluorescence output in our study depended on both the uptake of the plasmid and the gene expression efficiency. Therefore, the actual uptake efficiency of the plasmid should be higher than what we have estimated.

We tested several plasmas and found that Ar or Ar + H_2_O plasma had the best effect on cellular uptake (Fig. 2). The addition of O_2_ and H_2_O in Ar plasma may produce more gaseous ROS, and O_2_ and H_2_O may also reduce plasma intensity as they are electron-negative gases. We therefore measured the aqueous ROS after various plasma treatments in the medium and found that Ar or Ar + H_2_O plasma had a higher aqueous ROS level than other treatments (Fig. 4). Ar plasma showed similar aqueous ROS generation despite the lack of gaseous ROS compared to Ar + H_2_O plasma, mainly because the greater extent of gas ionization of Ar could partly convert into aqueous ROS. We propose that it is the aqueous ROS rather than the gaseous ROS that is correlated with the effects on plasma transfection. This mechanism is different from the previous standard transfection techniques that used electric breakdown and liposoluble packages. ROS accumulation-induced transfection appears to be gentle and efficient with less cell death than previous methods, which might allow for its development as a common tool for cell transfection of oligonucleotides. As shown in Fig. 4, a short duration of plasma treatment of cells did not increase the aqueous ROS levels as cells may react with exogenous ROS produced by plasma. This finding also indicates that the cell viability was not affected because the ROS accumulation was still within the tolerance levels. Yet, the intracellular ROS level was elevated after 20 s of plasma treatment (Fig. 6), although the aqueous ROS level in the medium remained the same. We further detected the intracellular Ca^2+^ level, and the results indicated that plasma treatment for 20 s increased cell membrane permeability as Ca^2+^ influx was observed after treatment (Fig. 7). Strangely, microscopy data showed a decrease in the fluorescence after 3 min of treatment (still higher than in the control), which was inconsistent with the cytometry data. Three minutes is a long duration of treatment, which results in serious damage to the cell membrane and cause cell death by viability assay (data not shown). The fluorescence of these cells could be detected by cytometry. However, the cells need to be centrifuged to the slide for 9 min before fluorescence microscopy. Part of the fluorescence may flux out of the cells, and the cells may even crash during this process, resulting in lower fluorescence after 3 min of treatment as detected by fluorescence microscopy. That is also the reason that we used a low dose of plasma treatment (20 s) for transfection in our study. Furthermore, depleting the aqueous ROS by several ROS scavengers would reduce the intracellular ROS level and weaken Ca^2+^ influx, resulting in a lower plasma uptake efficiency (Fig. 8). These data suggest that ROS are involved in the improved efficiency of plasma transfection. In our study, the plasma jet itself was grounded; thus, the effect of the electric field was limited. Chelsea *et al*. recently reported that using the plasma-activated air could deliver plasmid DNA into cells^29^, also suggesting that ROS are important for plasma transfection as the electric field is not involved in this process. However, how do the extracellular ROS produced by plasma transport into cells needs to be concerned. Small molecules such as H_2_O_2_ can catalytically pass through the cell membrane with the aid of the membrane transporters, aquaporins^43^,^44^. However, other ROS, such as O_2_^−^ and OH^−^ radicals, cannot pass through the cell membrane owing to negative charge and high reactivity, respectively. How they interact with cells and result in the accumulation of intracellular ROS remains unknown. As ROS scavengers could only partially inhibit plasma uptake, it is likely that intracellular ROS accumulation may be one of the mechanisms for plasma transfection. Other factors, such as charged particles and electric current produced by plasma, may contribute to plasma-mediated transfection. These factors need to be further investigated.

Finally, we expanded plasma transfection of DNA-FITC to other molecules, such as siRNA, miRNA and plasmids in 2D and 3D culture conditions. It seems that the effects on plasma transfection were partly dependent on the molecule size as siRNA were delivered into cells more efficiently than plasmids (Fig. 9). Furthermore, we showed that plasma transfection could be used for siRNA and miRNA experiments, especially in a 3D culture model. Although the transfection in 3D cultured system needs to be optimized and improved, plasma transfection provides a unique potential to transfect siRNA and miRNA directly into 3D cultured cells. Numerical and experimental data^45^,^46^,^47^ showed that reactive oxygen and nitrogen species generated by gas discharge plasmas are capable of penetrating 40–60 μm into an aqueous environment. Taken together with the evidence of ROS-mediated transfection in 2D and 3D models, these data suggest a deeper penetration of gaseous ROS/RNS into the aqueous environment, which is consistent with the finding of a gelatin-based tissue model^41^ and the possibility of *in-situ* ROS generation in the presence of cells^48^. Thus, trying to deepen the plasma penetration of reactive species in a 3D culture system could be a promising strategy to optimize and improve plasma transfection.

## Conclusions

In conclusion, we demonstrated that cold atmosphere plasma could be used as an efficient tool for delivering oligonucleotides. Aqueous extracellular ROS production by plasma is likely to be one of the primary factors underlying the efficacy of plasma transfection by increasing the intracellular ROS level and consequently changing the cell membrane permeability. In addition, we found that plasma could transfer siRNA and miRNA into cells even in 3D culture conditions. Our data suggest the possibility of transferring siRNA and miRNA by cold atmosphere plasma in 2D or 3D cell culture conditions.

## Materials and Methods

### Plasma production and characterization

The cold atmospheric plasma used in this study was generated by a plasma jet system which is described in our previous studies^48^. It is consisting of a high-voltage AC power supply, gas source, gas flow controller, and oscilloscope as well as the plasma jet device. The plasma jet has a 1.0 mm powered tungsten needle enclosed in a quartz tube and a grounded outer electrode wrapped around a 6.0 mm diameter dielectric tube^48^. The working condition is 2 SLM Ar gas flow with a power supplied at 10 kHz/10 kV.

### Cell culture conditions

We utilized the LP-1^49^ multiple myeloma cell line in this study. LP-1 cells were kindly donated by doctor Hu from the Department of Molecules and Genetics, medical school of Xi’an Jiaotong University. These suspension cells were grown in Roswell Park Memorial Institute (RPMI) 1640 medium supplemented with 10% fetal calf serum, 100 U/mL penicillin, and 50 μg/mL streptomycin (Corning, Ithaca, NY, USA). Cells were cultured at 37 °C in an incubator (Thermo Scientific Varioskan Flash, Waltham, MA, USA) containing 5% CO_2_. Cells were refreshed 24 h before performing experiments.

### Plasma transfection

For transfection, 1 × 10^5^ Cells were seeded evenly in a 24-well plate in 300 μL of RPMI1640 medium. The distance from the plasma jet to the liquid level was 1.5 cm. Cells were treated for 20 s by Ar plasma with different gas mixtures (N_2_, O_2_, and H_2_O), and 0.2 μg DNA-FITC was added in the medium for transfection. DNA-FITC (purchased from Sangon, Shanghai, China) is a chemically synthesized ssDNA (sequence: tgacgtgattccgtgaacca) with the fluorescence marker FITC added to the 5′ end of the DNA and is abbreviated as DNA-FITC in the following discussion. Cells were further cultured for 24 h and harvested. Cells were washed with 1mLphosphate buffered saline (PBS) solution 3 times and transfection was evaluated by flow cytometry and fluorescence microscopy. The control group received the same amount of DNA-FITC and the same gas flow but without discharging of plasma.

### Optical emission spectroscopy

The emission spectra of the plasma were measured using a UV/Visible spectrometer (Maya pro 2000, Ocean Optics, China) within a wavelength range of 280–400 nm. The emission spectra of Ar plasmas with different gas mixtures (N_2_, O_2_, and H_2_O) were analyzed in the vertical direction of the plasma jet. Because the intensity of the light is highest the moment that the plasma flows into the air, the optical probe was mounted at the nozzle of the plasma jet generator, which guarantees a clear spectrum of the particles in the plasma plume.

### Aqueous ROS detection

We measured the general ROS level and H_2_O_2_ concentration in the medium after various plasma treatments. 300 μL RPMI1640 medium was added to 24-well plates with or without LP-1 cells (1 × 10^5^/well) and was treated with different plasmas for 20 s and 40 s. 100 μL medium was collected for the measurement of general ROS level and H_2_O_2_ concentration at 0 h, 6 h and 18 h after the treatment. ROS level was measured by CM-H2DCFDA, according to the protocol by Ishaq^40^,^41^. Briefly, 0 h, 6 h and 18 h after plasma treatment, 100 μL of the medium were incubated with 10 μM of CM-H2DCFDA (Invitrogen, Carlsbad, CA, USA) (dissolved in DMSO at 10 mM) for 30 min at 37 °C in the dark. ROS were measured by a microplate reader (Thermo) at excitation and emission wavelengths of 485 and 530 nm, respectively, using the protocol for fluorometric measurement. H_2_O_2_ concentration was measured by Amplex Red Hydrogen Peroxide Assay (Invitrogen), as described in detail in our previous study^48^. Briefly, 0 h, 6 h and 18 h after plasma treatment, 100 μL of the medium were incubated with 50 μM of Amplex^®^ Red reagent and 0.1 U/ml of Horseradish peroxidase (HRP) for 30 min in the dark. Fluorescence was monitored at excitation and emission wavelengths of 530 and 590 nm by a microplate reader (Thermo)using the protocol for fluorometric measurement. A gradient H_2_O_2_ standard curve was generated to calculate H_2_O_2_ concentration.

### ROS scavengers

Several ROS scavengers were used to distinguish the component efficacy in the plasma. These scavengers were purchased from Sigma-Aldrich (St. Louis, MO, USA), including sodium pyruvate for H_2_O_2_^50^; mannitol for OH^50^; tiron for O^2^,^3^,^4^,^5^,^6^,^7^,^8^,^9^,^10^,^11^,^12^,^13^,^14^,^15^,^16^,^17^,^18^,^19^,^20^,^21^,^22^,^23^,^24^,^25^,^26^,^27^,^28^,^29^,^30^,^31^,^32^,^33^,^34^,^35^,^36^,^37^,^38^,^39^,^40^,^41^,^42^,^43^,^44^,^45^,^46^,^47^,^48^,^49^,^50^,^51^; and N-acetyl cysteine (NAC) as a general ROS scavenger^52^,^53^. These scavengers are specific with little cross-reactivity to other ROS, so they are used in many protocols for investigating particular ROS functions. The scavengers were added prior to plasma treatment to guarantee their effectiveness at a final concentration of 10 mM for sodium pyruvate; 50 mM for mannitol; 10 mM for tiron; and 20 μM for NAC. In addition, to test whether the UV in plasma is functional, we used a quartz plate to block all the reactive species except the UV that could pass through the plate and reach the cells.

### Transfection via lipofection

For lipofection, 1 × 10^5^ cells were seeded in a 24-well plate in 300 μL RPMI1640 medium. Then 2.5 μL of Lipofectamine 2000 reagent (Invitrogen) and 0.2 μg DNA-FITC were diluted separately into 50 μL serum-free medium and mixed together for 5 min. The DNA-lipid mixture was added into the cells and further incubated for 24 h. The same amount of DNA-FITC without lipofectamine 2000 was used as a control. Cells were harvested and washed with 1 mL PBS 3 times. The uptake efficiency was detected by flow cytometry and fluorescence microscopy.

### Transfection via electroporation

For electroporation, 4 × 10^5^ cells were harvested and resuspended in 200 μL PBS in 0.2 cm cuvettes. Then, 0.2 μg of DNA-FITC was added to each sample for electroporation. Two electroporation conditions were used for the transfection following the operator manual by Gene PulserXcell (BD, Franklin Lakes, NJ, USA):1) electroporation 150 V, 10.0 ms pulse length, 1 pulse number, and 2 mm cuvette; and 2) electroporation 160 V, 500 μF capacitance, ∞ resistance, and 2 mm cuvette. After electroporation, cells were washed with 1 mL RPMI1640 medium and transferred to a 6-well plate in 2 mL medium for culture. After 24 h, cells were washed with 1 mL PBS 3 times and the transfection efficiency was determined by flow cytometry and fluorescence microscopy. The same electroporation procedure without adding DNA-FITC was used to determine the corresponding cell apoptosis by Annexin-V/PI staining.

### Cell viability assay

A CellTiter-Glo assay (Promega, Madison, WI, USA) was used to assess cell viability. This assay is a luminescent test based on the quantification of ATP. Because the level of ATP represents the metabolic activity of the cell, luminescence intensity is positively correlated to the number of viable cells. Twenty-four hours after transfection, 100 μL of cell suspension were added to 100 μL of luminometric reagent in a 96-well non-transparent plate. To fully induce cell lysis, the 96well plate was placed on an orbital shaker for 2 min and the cells were then incubated at room temperature for 10 min. The luminescence intensity was recorded using the microplate reader (Thermo) using the protocol for luminometric measurement (the measurement time was set to 1000 ms without choosing a wavelength).

### Annexin-V and PI staining

The apoptosis of LP-1 cells after transfection was detected by flow cytometry using an Annexin-V/PI apoptosis kit (BD). After transfection for 24 h, cells were harvested and washed twice with Dulbecco’s PBS without calcium and magnesium (Corning). Cells were resuspended in 50 μL 1× binding buffer (0.01 M Hepes/NaOH (pH 7.4), 0.14 M NaCl, 2.5 mM CaCl_2_) with 2 μL annexinV-APC (3 μg/mL) and 2 μL PI (50 μg/mL) and incubated at room temperature in the dark for 15 min. An additional 400 μL 1× binding buffer was added, and samples were analyzed by flow cytometry.

### Intracellular ROS level detection

The fluorescent probe DCFH-DA was used to detect intracellular ROS levels. DCFH-DA can independently pass through the cell membrane and can be deacetylated to DCFH by esterase. Once deacetylated, DCFH cannot cross the cell membrane again and becomes deposited in the cells. Because ROS can oxidize non-fluorescent DCFH to fluorescent DCF, the fluorescence intensity is correlated with the intracellular ROS level. DCFH-DA (Sigma) was dissolved in DMSO at a concentration of 20 mM as a stock solution. Then, 1 × 10^6^ cells were suspended in 1 mL PBS with 20 μM of DCFH-DA and incubated for 30 min at 37 °C. Samples were washed with 1 mL PBS 3 times and fluorescence was measured by flow cytometry (with green fluorescence channel, FL1) or fluorescence microscope using the blue filter (490–505 nm) for excitation and the green filter (515–545 nm) for emission.

### Intracellular Ca^2+^ level detection

Cells were harvested and washed with Hank’s Balanced Salt Solution (HBSS) 3 times. Cells were incubated with 2 μM Fluo 3-AM (Sigma) for 30 min at 37 °C with gentle shaking every 5 min to blend the dye. Cells were washed with HBSS 3 times and further incubated for 30 min at 37 °C to ensure that the Fluo 3-AM in the cells was fully esterified into Fluo 3. Cells were washed with 1 mL PBS 3 times, resuspended in PBS and fluorescence was measured by flow cytometry and fluorescence microscopy.

### Flow cytometry

Fluorescence was detected by an Accuri C6 flow cytometry (BD). Samples were collected and washed with 1 mL PBS 3 times and resuspended in PBS at a concentration of 2 × 10^5^/mL. The primary threshold of FSC-H was set to 800000 to eliminate small impurities. According to FSC-A and SSC-A parameters, we used the normal LP-1 cells to gate the cell population, that is, to exclude the cell debris. Ten thousand events were acquired for each sample. For FITC, DCFH-DA and Fluo-3AM, we used FL1 green channel to detect the fluorescence. The corresponding fluorescence of untreated cells was used as a control to gate the fluorescence positive cells. The uptake efficiency was calculated as the percentage of the fluorescence positive cells. For the analysis of PI and APC in apoptosis, we used the FL2 yellow channel and FL4 red channel, respectively. Single staining of PI and APC positive samples was used to correct the compensation. Little fluorescence overlap was found between PI and APC, so no compensation was needed in our study.

### Fluorescence microscopy

After transfection, 300 μL of LP-1 cells were collected and washed with 1 mL of PBS 3 times. Cells were resuspended in 200 μL PBS at a concentration of 4 × 10^5^cells/mL. Cells were centrifuged onto a microslide at 200 × *g* for 9 min using a CytoSpin instrument (Wescor 7621, Logan, UT, USA). Fluorescence was photographed and analyzed using an epifluorescence microscope (BX53 and DP73, Olympus, Tokyo, Japan). To detectgreen fluorescence such as DNA-FITC, DCF and Fluo-3AM, we used the blue filter (490–505 nm) for excitation and the green filter (515–545 nm) for emission. Image Pro Plus 6.0 (IPP) software was used to measure the fluorescence intensity. For each sample, we randomly selected 10 equal areas (AOI) and automatically chose the cells for measurement. We calibrated the intensity to Std. Optical Density model and the mean of the integrated option density (IOD) was calculated for analysis.

### Plasma transfection of DNA-FITC, siRNA-FITC, miRNA and pEGFP-C1 plasmid in 2D and 3D culture conditions

For the 2D culture condition, 1 × 10^5^ cells were plated in 300 μL RPMI1640 medium in a 24-well plate. Cells were treated for 20 s by Ar plasma and 5 μL of DNA-FITC (0.2 μg), siRNA (0.5 μg), miRNA (0.5 μg) or pEGFP-C1 (0.1 mg) were added to the medium for plasma transfection. For the 3D culture condition, LP-1 cells were refreshed and resuspended in 50 μL RPMI medium at a concentration of 2 × 10^6^/mL. Cells were mixed with 0.3 mL Matrigel basement membrane matrix (Corning) and plated in 24-well dishes for 3D culture. After 24 h, cells were treated with Ar plasma for 40 s and 20 μL of DNA-FITC (0.8 μg), siRNA (2.0 μg), miRNA (2.0 μg) or pEGFP-C1 (0.4 mg) was added to the plate for plasma transfection. After another 24 h, 10 mL RPMI medium was mixed with a 3D matrix to dissolve the matrix by blowing and beating repeatedly. Cells were centrifuged at 800 rpm for 3 min and the suspension was discarded. This process was repeated 2 times before the cells were resuspended in 500 μL PBS. The uptake efficiency was detected by flow cytometry using the green fluorescence channel. siRNA-FITC (sc-36869, 19 bp) was purchased from Santa Cruz Biotechnology (Dallas, TX, USA), and miRNA (has-miR-200b, #4464066) was purchased from Life Technologies, Carlsbad, CA, USA. pEGFP-C1 was purchased from YouBio company, Changsha, China.

### Western blotting

Cell pellets were lysed in lysis buffer containing 50 mM Tris, 150 mM NaCl, 1% Nonidet P40, and 0.25% sodium deoxycholate. Cell debris was removed by centrifugation for 5 min at 4000 × *g* before sample buffer was added. After boiling, samples were separated by sodium dodecyl sulfate-polyacrylamide gel electrophoresis (SDS-PAGE) and transferred to polyvinylidene difluoride membranes (Bio-Rad, Hercules, CA, USA), which were blocked with PBS containing 5% low-fat milk and 0.1% Tween 20. Membranes were probed with antibodies against human ferritin heavy chain (FTH1) (1:1000) and *β-actin* (1:1000) (Cell Signaling Technology, Danvers, MA, USA). Membranes were washed with PBS containing 0.1% Tween 20 (PBST) for 30 min and then incubated with horseradish peroxidase-conjugated goat anti-rabbit IgG (1:2000 for FTHI) and anti-mouse IgG (1:2000 for β-actin) for 30 min at room temperature. Membranes were washed in PBST and imaged using a ChemiDoc-It 510 system (UVP, Upland, CA, USA).

### Statistical analysis

All experimental conditions were prepared in triplicate and experiments were repeated at least three times. Data are presented as means ± SD. Differences between groups were evaluated using the Mann-Whitney U test. P < 0.05 was considered statistically significant.

## Additional Information

**How to cite this article**: Xu, D. *et al*. Intracellular ROS mediates gas plasma-facilitated cellular transfection in 2D and 3D cultures. *Sci. Rep.* **6**, 27872; doi: 10.1038/srep27872 (2016).
